# An Educational Evaluation of Medical Students' Attitudes Towards Intellectual Disability

**DOI:** 10.1111/tct.70214

**Published:** 2025-09-25

**Authors:** Maja Donaldson, Molly Hebditch, Nina Arnesen, Penny Geer, James Fallon, Sarah Stringer, Stephanie Daley

**Affiliations:** ^1^ Sussex Partnership NHS Foundation Trust Millview Hospital Hove East Sussex UK; ^2^ Brighton and Sussex Medical School University of Sussex Falmer East Sussex UK

**Keywords:** evaluation, intellectual disabilities, learning disabilities, medical education, medical students

## Abstract

**Background:**

There are 1.5 million people with intellectual disability living in the United Kingdom. It is recognised that people with intellectual disability are a marginalised group whose health experiences and outcomes are lower than those without intellectual disabilities. A contributing factor is a lack of knowledge and skills in the medical workforce.

**Approach:**

One medical school in England sought to address this challenge by developing an Intellectual Disability Training session for fourth‐year medical students.

**Evaluation:**

We sought to assess the impact of the new Intellectual Disability Training session on student attitudes towards people with intellectual disability and student satisfaction with the session. All students were invited to take part in this evaluation prior to completion of the mandatory Intellectual Disability Training. Students were asked to a complete a pre‐post attitude questionnaire and a satisfaction survey. One hundred eighty students participated in the evaluation out of a cohort of 210 students. Paired outcome data were collected for 113 students. A significant increase in attitude scores was found in four of the five factors (discomfort, emotional, knowledge of capacity/rights and behaviour). Feedback from the session has identified positive aspects, as well as areas for development.

**Implications:**

This evaluation has identified that an Intellectual Disability Training session can positively impact student attitudes towards people with intellectual disabilities. Such programmes could be implemented more widely at undergraduate level to enhance the future care delivery to this marginalised group of people.

## 1 Background

People with intellectual disability are likely to have complex health needs, making them high users of healthcare services [1]. The 2022 Learning Disabilities Mortality Review (LeDeR) report shows that people with intellectual disability have, on average, a 20‐year lower life expectancy, and significantly higher rates of excess deaths, than the general population [2]. A number of barriers to accessing healthcare have been identified as contributors, including a lack of knowledge and understanding in the workforce [3].

*The 2022 Learning Disabilities Mortality Review (LeDeR) report shows that people with intellectual disability have, on average, a 20‐year lower life expectancy…*.

In England, it has recently become mandatory for healthcare providers to ensure the workforce has adequate training in intellectual disability [4]. While this does not cover medical students, it is apparent that medical training is an optimal time for such teaching. Intellectual disability teaching for undergraduate medicine varies significantly, and studies vary in methodology and outcome, although intellectual disability‐specific teaching appears to have a positive impact, and changes in attitude are more likely through the involvement of lived experience [5, 6].

## 2 Approach

Brighton and Sussex Medical School in the South of England noted a sparsity of intellectual disability teaching across the academic programme on curriculum mapping. We addressed this systemic challenge through a pragmatic, emancipatory approach to allow students to understand the challenges individuals with intellectual disability face and empower their own practice [7].

An intellectual disability training session for fourth‐year medical students was developed by a group of educators to improve attitudes to people with intellectual disability. A single day in the timetable was identified, and subject experts were approached for collaboration. The three‐hour mandatory session was delivered in small groups, involving a mixture of didactic and interactive components (Table 1). Participants received theoretical grounding in healthcare inequalities and approaches to management for individuals with intellectual disability, including communication techniques. They were introduced to the role of different professionals specialising in intellectual disability, such as consultant psychiatrists and learning disability nurses. Using a constructivist approach to build on the didactic content [8], small group work covered case studies in primary and acute care settings and two tailor‐made films involving a subject specialist and people with an intellectual disability.

*An intellectual disability training session for fourth‐year medical students was developed by a group of educators to improve attitudes to people with intellectual disability*.

**TABLE 1:** Learning outcomes and content for the Intellectual Disability Training.

Learning outcomes for the Intellectual Disability Training
By the end of this session, students will be able to: • Describe common health conditions in people with learning disabilities. • Explain ways in which doctors can meet the physical health needs of people with learning disabilities and work towards reducing health inequalities. • Understand what is meant by diagnostic overshadowing and be able to identify key features of acute presentations of people with a learning disability. • Know how and when to seek assistance and advice from specialist services for people with learning disabilities in hospital and community practice.

Intellectual Disability Training session content						
Pre‐Session Questionnaire	Introductory talk: Healthcare for individuals with intellectual disability	Video: Interview with subject expert	Small group work: Intellectual disability in primary care (health action plan)	Large group work: Worked case (acute presentation)	Video: Lived experience in conjunction with local charity	Post‐session Questionnaire

## 3 Evaluation

The aims of our evaluation were to

1. Assess the impact of the Intellectual Disability Training session on student attitudes towards people with intellectual disability, using a validated attitudes measure
2. Assess student satisfaction with the session to inform the structure of future iterations of the Intellectual Disability Training.

We used the Attitudes Towards Intellectual Disability (ATTID) short form scale [9] to obtain quantitative data before and after the Intellectual Disability Training session. The ATTID scale has five factors, shown in Table 2.

**TABLE 2:** Description of ATTID factors [10].

ATTID factors	Attitudinal dimension measured
Factor 1: Discomfort	Emotional dimension
Factor 2: Knowledge of capacity and rights	Cognitive dimension
Factor 3: Interaction	Behavioural dimension
Factor 4: Sensitivity or tenderness	Emotional dimension
Factor 5: Knowledge of causes	Cognitive dimension

*We used the Attitudes Towards Intellectual Disability (ATTID) short form scale to obtain quantitative data before and after the Intellectual Disability Training session*.

All fourth‐year students received information about the evaluation prior to, and at the beginning of the Intellectual Disability Training, and all students were invited to complete the measure before and after the teaching session. The pre‐session questionnaire (T1) included the ATTID scale supplementary questions. The post‐session (T2) questionnaire included feedback questions about the session and a voucher incentive. Student data between pre‐ and post‐session was matched and anonymised.

Quantitative data were analysed following the ATTID scale guidance [10], and the mean responses were used to run a paired sample *t*‐test.

The qualitative data were analysed through conventional content analysis [11].

Ethical approval for this study was obtained from the Research Governance and Ethics Committee, University of Sussex.

One hundred eighty students (85%) out of 210 fourth‐year medical students attended the Intellectual Disability Training session and consented to participate in the study. Student characteristics are shown in Table 3.

**TABLE 3:** Participant characteristics.

Characteristic	Range	Number	(%)
Age range (years)	20–24	115	72.8
	25–29	36	22.8
	30–34	3	1.9
	35–39	4	2.5
	Missing	22	
Gender identity	Female	102	64.6
	Male	55	34.8
	Non‐binary	1	0.6
	Missing	22	
Ethnicity	White British/European	80	50.6
	Asian/Asian British	52	32.9
	Black/African/Caribbean/Black British	13	8.2
	Mixed/Multiple ethnic groups	5	3.2
	Other	4	2.5
	Arab/Middle Eastern	1	0.6
	Hispanic	1	0.6
	Kurdish	1	0.6
	Mixed	1	0.6
	Missing	22	
How much do you know about intellectual disability	Nothing	7	4.7
	Not much	98	65.8
	Quite a bit	42	28.2
	A lot	2	1.3
	Missing	31	
How many people with intellectual disability do you know or have met	0	15	10.1
	0–5	83	56.1
	5–10	16	10.8
	10–20	7	4.7
	20–30	6	4.1
	30–50	1	0.7
	50–100	2	1.4
	A lot	12	8.1
	Do not know	6	4.1
	Missing	32	

### 3.1 Quantitative Data

The ATTID scale consolidates responses into three groups: positive (scores of 1 or 2), neutral (scores of 3) or negative (scores of 4 or 5). Table 4 shows that the percentage of positive responses increased across Factors 1, 2, 3 and 4 following participation in the teaching session.

**TABLE 4:** Comparison of attitudes in T1 (pre‐session) and T2 (post‐session) for each factor.

Attitudes	Percentage of participants (%)									
	Factor 1 (discomfort)		Factor 2 (capacity/rights)		Factor 3 (interaction)		Factor 4 (tenderness)		Factor 5 (causes)	
	T1	T2	T1	T2	T1	T2	T1	T2	T1	T2
Positive	71.5	83.0	82.7	87.7	83.3	87.9	45.3	59.6	81.3	81.9
Neutral	14.4	10.4	12.4	8.8	11.5	9.2	28.0	23.0	11.9	12.1
Negative	14.1	6.6	4.9	3.5	5.2	3.0	26.7	17.5	6.8	6.0
Total	161	146	166	151	162	147	161	145	167	146

Table 5 presents the score for each factor at pre‐ and post‐session in terms of mean scores and standard deviation. In the ATTID scale, the lower the mean, the more positive the attitudes towards intellectual disability.

**TABLE 5:** Results of T‐tests (T1 = pre‐session, T2 = post‐session).

	Paired sample statistics				Matched pairs	T‐test		Effect size		
						*t*	Two‐sided *p*	Cohen's *d*	95% confidence interval	
		Mean	Std. deviation	Std. error mean					Lower	Upper
Factor 1: Discomfort	T2	1.737	0.754	0.065	135	−6.864	< 0.001	−0.591	−0.773	−0.407
	T1	2.054	0.838	0.072						
Factor 2: Knowledge of capacity and rights	T2	1.670	0.535	0.046	137	−3.642	< 0.001	−0.311	−0.482	−0.139
	T1	1.817	0.513	0.044						
Factor 3: Interaction	T2	1.621	0.519	0.046	130	−5.778	< 0.001	−0.507	−0.689	−0.323
	T1	1.808	0.570	0.050						
Factor 4: Emotional	T2	2.310	0.828	0.075	122	−7.072	< 0.001	−0.640	−0.834	−0.444
	T1	2.684	0.808	0.073						
Factor 5: Knowledge of causes	T2	1.867	0.617	0.058	113	−0.482	0.631	−0.045	−0.230	0.139
	T1	1.888	0.518	0.049						

The results of the paired T‐test are shown in Table 5. All factors apart from 5 (knowledge of causes) met the threshold for statistical significance (*p* < 0.05).

**FIGURE 1:**
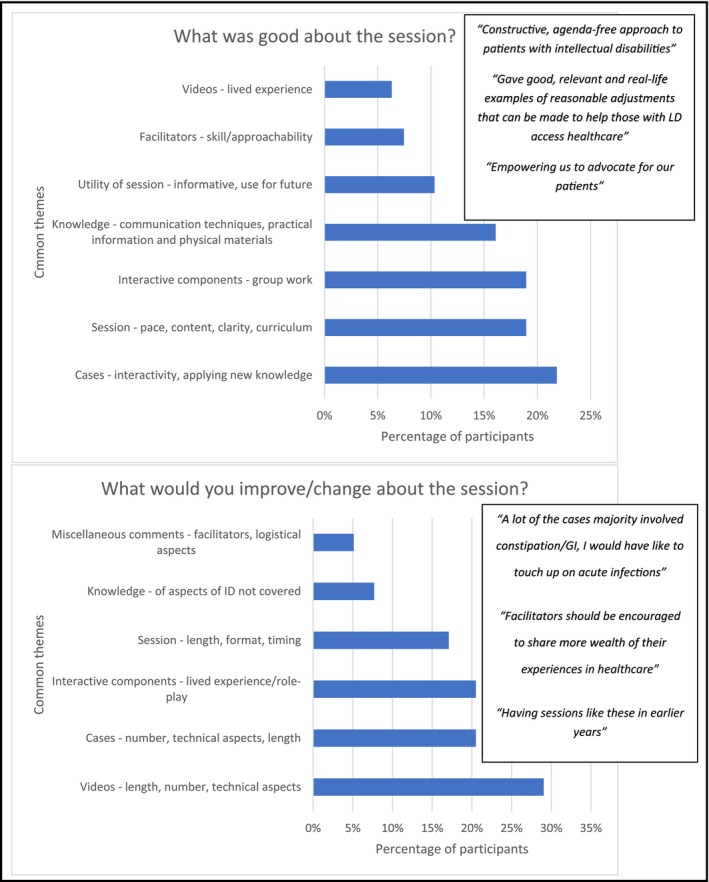
Content analysis of session feedback.

The post‐session survey is summarised below and in Figure 1.

The majority 99/180 (67.3%) of students felt that the session was the right length, with the remainder 48/180 (32.7%) reporting it was too long. Responses indicated that the majority of participants strongly agreed/agreed that the session was relevant to their curriculum 138/145 (95.2%) and their future career 141/145 (97.2%), it was well structured 129/146 (88.4%) and enjoyable 127/148 (85.8%), and 138/148 (93.2%) of respondents felt more confident working with individuals with intellectual disability after the Intellectual Disability Training.

In terms of positive feedback, participants liked the cases, interactive components and exposure to lived experience through videos. They felt the session would be useful for the future, and left them feeling empowered to advocate for patients. Participants also commented on the content: variety, being well‐structured and inclusive.

Regarding improvements, participants felt the session and videos were both too long. They wanted more interactive and knowledge components, and greater inclusion of lived experience.

## 4 Implications

The evaluation suggests that the Intellectual Disability Training produced a significant, positive shift in student attitudes towards intellectual disability, most significant in the affective and behavioural dimensions. Knowledge of capacity and rights did show a significant positive change, while knowledge of causes did not show any significant change. To measure the reliability of the Intellectual Disability Training in affecting attitudes, the teaching team conducted a similar cycle of data collection in the second iteration. Across two iterations, the Intellectual Disability Training has resulted in a statistically significant shift towards more positive attitudes post‐session.

*The evaluation suggests that the Intellectual Disability Training produced a significant, positive shift in student attitudes towards intellectual disability…*.

In assessing student satisfaction, students particularly valued interactivity, the case studies, and there was a wish for greater inclusion of lived experience. This aligns with literature noting that face‐to‐face interactions with people with intellectual disability brought about a statistically significant change in attitudes [5]. It is likely that higher satisfaction with teaching will enhance openness and willingness towards attitudinal change and thus address the aim of the Intellectual Disability Training programme.

*In assessing student satisfaction, students particularly valued interactivity, the case studies, and there was a wish for greater inclusion of lived experience*.

In planning session‐related changes for future iterations, educators were guided by qualitative feedback, which aligned with informal feedback from facilitators following the session. Ideally, this would have involved focus groups, but this was not within the scope of the evaluation. A number of themes were identified, including the content and values of the session and logistical components. We continued to adopt a mixture of didactic and interactive approaches to the presentation of material and Freire's [7] emancipatory approach in the second iteration, transgressing the boundaries between teacher, student, and patient [12].

The second iteration started with a large group format to accommodate a question and answer session with a subject expert and a lived‐experience talk with a local charity. Preliminary results from the second iteration indicate this was well received by students, which supports works cited in showing how impactful lived experience can be on student understanding and learning. The case‐study work remained in small groups to facilitate interactivity and allow reflection on the large‐group session.

Key lessons learnt during the process of forming and altering the Intellectual Disability Training fall broadly into categories of format, content and logistics. Logistically, a large team of passionate individuals with a broad range of skills enabled us to pragmatically structure the session within a limited deadline. With session format, the value of minimising didactically conveyed information was reflected in student feedback following the second iteration. Regarding content, an emphasis on practical information and lived experience was well received by students in both iterations. These points are salient considerations for other educators considering delivering a similar session on intellectual disability.

Regarding local change, the Intellectual Disability Training moves towards ensuring the medical school curriculum mirrors changes in legislation for intellectual disability training and addressing the recommendations made in guidance made by specialty bodies [13, 14]. Although this study focussed on medical students, the programme would ultimately be pertinent for students from any healthcare discipline. This could be a consideration for future iterations, as the local university runs other healthcare training programmes.

*… the Intellectual Disability Training moves towards ensuring the medical school curriculum mirrors changes in legislation for intellectual disability training …*.

## Author Contributions

**Maja Donaldson:** investigation, data curation, formal analysis, writing – original draft. **Stephanie Daley:** investigation, conceptualization, data curation, formal analysis, writing – original draft. **James Fallon:** investigation, conceptualization, data curation, writing – review and editing. **Nina Arnesen:** investigation, conceptualization, data curation, writing – review and editing. **Penny Geer:** investigation, conceptualization, data curation, writing – review and editing. **Sarah Stringer:** conceptualization, data curation, writing – review and editing. **Molly Hebditch:** formal analysis, writing – original draft.

## Ethics Statement

Ethical approval for this study was obtained from the Research Governance and Ethics Committee, University of Sussex.

## Consent

Written consent from taken from student participants at the beginning of the teaching session. Information about the study was sent to all potential student participants 1 week before.

## Conflicts of Interest

The authors declare no conflicts of interest.

## Data Availability

Data are available from the corresponding author on reasonable request.
